# Supplementary material for: Making the BEST decision-the BESTa project development, implementation and evaluation of a digital Decision Aid in Swedish cancer screening programmes- a description of a research project
Source: PLoS One. 2023 Dec 12;18(12):e0294332. doi: 10.1371/journal.pone.0294332 (PMC10715660; doi:10.1371/journal.pone.0294332)
Supplement: S1 Fig — (DOCX) [file pone.0294332.s002.docx]

Supporting information S2. An overview of the process, phase 1
