# Supplementary material for: Making the BEST decision-the BESTa project development, implementation and evaluation of a digital Decision Aid in Swedish cancer screening programmes- a description of a research project
Source: PLoS One. 2023 Dec 12;18(12):e0294332. doi: 10.1371/journal.pone.0294332 (PMC10715660; doi:10.1371/journal.pone.0294332)
Supplement: S2 Fig — (DOCX) [file pone.0294332.s003.docx]

Supporting information S3. Self-reported questionnaires phase 2

**Questions when entering the decision aid:**

**Do you visit BESTa**

- For yourself
- for somebody else
- because you need a follow up examination

**Optional interactive questions/statements to increase awareness:**

- **values/preferences,** e.g. **”cancer** screening is beneficial for me”, “I believe that others benefit from cancer screening”
- **understanding/knowledge**, e.g. ”I understand the benefits and disantvantages of cancer screening”, ”I’m unsure about participation in cancer screening”
- **lifestyle,** e.g. “it is important to me to be physical active/eat healthy to stay healthy”, “do you smoke”

**Questions that appear when consent to data saving:**

**demography**

- gender
- age
- living situation
- education
- occupation

**Questions/statements when individuals leaving the decision aid:**

**Evaluation of the decision aid**

- ”my understanding (knowledge) about cancer and screening increased which is beneficial for me”
- “using the decision aid helped me to make a satisfying decision about participation in cancer screening”
- “I will recommend the decision aid to others who are interested in cancer and screening”
- digital literacy, e.g. ”there were text/images/numbers included in the decision aid that were difficult to comprehend
