# Supplementary material for: Making the BEST decision-the BESTa project development, implementation and evaluation of a digital Decision Aid in Swedish cancer screening programmes- a description of a research project
Source: PLoS One. 2023 Dec 12;18(12):e0294332. doi: 10.1371/journal.pone.0294332 (PMC10715660; doi:10.1371/journal.pone.0294332)
Supplement: S1 File — (DOCX) [file pone.0294332.s004.docx]

**Title of main project:**

Towards high, equal and informed participation in Swedish cancer screening – the BESTa

project.

**Subtitle of present project:**

Development and implementation of a Decision Aid in Swedish cancer screening programs

**OBJECTIVES**

For breast-, cervix- and bowel cancer screening programs to be effective a high willingness,

of seemingly healthy individuals to participate is a prerequisite. Participation rates differ

between programs and regions and are relatively high in some population groups, but at the

same time low in other groups. To apply an equity perspective on screening, it is desired that

individuals make an informed decision on knowledge rather than ignorance, misconceptions

or fear. Hence in terms of equity applying a gender- and diversity perspective is crucial for the

present project and in line with more or less three of the sustainable development goals: Good

health and well-being, Gender equality and Reduced inequalities, all included in Agenda 2030

decided by UN in 2015 and communicated by our government and authorities. [1, 2]. Results

from our previous findings and the present project will form the basis for an intervention with

a web-based decision aid (DA), to be implemented in all cancer screening programs starting

with bowel cancer, followed by breast- and cervical cancer. The web-based DA will be

accessible online and with various content and directed towards individuals approached to

cancer screening, with the goal of supporting informed decision making. The advantage of a

DA is that individuals gain knowledge of cancer and screening entering one webpage with a

possibility to communicate for advice and support with health professionals, instead of

searching the internet. Existing evidence show that people using DAs in connection to a

treatment- or screening decision increase their knowledge and they feel more informed and

certain of what matters most to them [3, 4]. DAs can also be cost-effective, as shown by a

decision-analytic model [5]. Still, more research is warranted and no DA for any of the

screening programs exist in Sweden. By designing the communication strategies in various

ways, regarding information about options, to help individuals to construct, clarify and

communicate personal values our hope is that the gender- and diversity perspective can be

addressed and acknowledged.

**Overall aim**

To develop and implement a web-based decision aid for individuals invited to cancer

screening in Sweden.

Specific aims based on the template from Coulter et al. [6]:

1. defining scope and purpose of the decision aid and target audience

2. assemble a steering group including clinical experts a lay people

3. designing the decision aid including lay people and clinician views, format, setting,

timing, clinical evidence, prototype development

4. alpha testing including comprehensibility and usability

5. beta testing including feasibility

6. DA evaluation of usability including chat function and helpline, behavior flow,

literacy, visitors’ knowledge, values and preferences

**RESEARCH OVERVIEW**

Sweden has a long tradition of organized national population-based screening programs. The

first started in the 60s with offering pap smear test to prevent cervix cancer, followed by

mammography screening for breast cancer in 1986 and a regional screening program with

fecal test for bowel cancer in 2008. The cervix cancer screening program includes women

aged 23 – 64 and since 2015, the National Board of Health and Welfare [7] recommends test

for human papillomavirus (HPV) every third year in women aged 23 – 49 and every seventh

years in women aged 50 – 63. In addition, self-sampling test kits for HPV have been offered

in recent years and even more so due to the COVID-19 pandemic. The breast cancer

screening program in Sweden offers mammography every other year for women aged 40 – 74.

For bowel cancer, screening has only been offered to citizens in the Stockholm – Gotland

region but are now to be introduced in all regions and expected to be fully implemented

during 2022 [8]. All screening programs in Sweden are free of charge. However, for a cancer

screening program to be effective, a high participation rate is necessary still, screening

programs are well known for facing challenges in obtaining a high participation rate and

thereby jeopardizing the benefits of screening, i.e., decreased morbidity. Moreover, a low

participation rate also jeopardize the equity of screening programs, since it is well known that

individuals with lower socioeconomic status, ethnic minorities and individuals with

disabilities participate to a limited extent in cancer screening [9-13]. A high participation rate

is therefore crucial, not only for preventing cervical-, breast- and bowel cancer in the

population, but also to reduce inequalities related to health prevention efforts, in this case

population-based screening. However, when seemingly healthy individuals are approached, it

is vital to ensure their autonomy [14]. In addition to socioeconomic factors, other reported

barriers irrespective of cancer screening program are poor understanding and knowledge of

cancer and screening [15-19]. Differences in barriers and facilitators for uptake between the

programs also relate to the screening method. For cervical cancer screening the use of selfsampling

HPV test has been found to be highly acceptable [20] and to increase uptake [21]. In

addition, when changing fecal sampling test to a more user-friendly sampling tube in bowel

cancer screening programs the uptake increased [22]. On the contrary, for breast cancer

screening, research shows that information interventions about the relatively high risk of over

detection with mammography, impacts the willingness to participate in a negative way [23].

**PROJECT DESCRIPTION**

***Design***

This study has a mixed-method approach based on the framework from IPDAS [24] and the

later update and revision by Coulter et al. [6].

***DA intervention***

Based on our previous research and relevant scientific literature, a DA (accessible online with

various content), will be developed towards individuals approached to cancer screening. DAs

are set to deliver information about different health care options and to help individuals

construct, clarify and communicate personal values connected to the options available. DAs

are not meant to guide individuals to choose one option over the another [25]. DAs in

screening usually include information on disease and screening, screening tests, benefit and

harms and some values clarification exercise (e.g. interactive questionnaires) to facilitate what

decision best matches the individuals´ values [3]. The DA will address all three screening

programs with one module for mutual content, and three separate modules for contents that

differ in cervix-, breast- and bowel cancer screening. We will start with the bowel cancer

screening module.

***Procedure***

Phase 1. The procedure will follow the template for documenting DA development process

proposed by Coulter and colleagues [6] using the following steps:

1. Define scope – information will be gathered regarding breast, cervix and bowel cancer

and treatment, screening tests, true positive true negative results, risks and benefits

with screening vs. non-screening, detection probabilities, describe possible next step

based on test results.

2. Steering group – a multidisciplinary group will be formed with stakeholders: lay

people (aged 23–75, different socioeconomic status and ethnicity, from urban and

rural areas, invited to and not invited to screening); clinical expertise (oncologists,

endoscopists, gastroenterologist, gynecologists, nursing expertise, psychologists) and

other experts (in psychometry and IT); collaboration partners (The Swedish

Association of Local Authorities and Regions - SKR, Regional Cancer Centers –

RCC, National Board of Health and Welfare – SoS).

3. Design – eliciting lay people and clinical experts’ views about the format (provision of

information and descriptions in text, images, audios, animated videos, certain

clickable words to provide additional information, and interactive questionnaires to

help individuals to clarify and express values); setting (web-based) and timing (link

will be provided with the invitation to cancer screening but also be open for everyone).

The design process will be documented regarding the quality of evidence that the DA

is based on and the description of the prototype development.

4. Alpha testing – the comprehensibility and usability of the developed DA will be

reviewed by lay people and clinical experts using qualitative methods (focus group

discussions, individual interviews in think aloud format).

5. Beta testing – lay people and clinical experts will test the feasibility of the decision aid

in a “real world setting” and it will be evaluated with a qualitative methods (focus

group discussions, individual interviews, think aloud).

For an overview of phase one, se figure 1.

Phase 2. After Beta testing the DA will be public and accessible for all who are invited to the

cancer screening programs for breast-, cervix-, and bowel as well as for others who are

interested. The DA will be evaluated by: 1/questions/statements to the users when they first

enter the DA, such as ‘do you enter the program because of: own interest/interest of

others/testing positive for blood in stool’, 2/ the content of the chat function and helpline; 3/

behavior flow; 4/questions/statements such as ‘the DA increased my knowledge of cancer and

screening/using the DA helped me to make a decision that I am satisfied with/the text was

easy to read/it took a long time to read the text/there where words I did not understand/there

where numbers I did not understand/there where images I did not understand/I felt

comfortable to respond to the questions/it was easy to find suitable response options. In

addition, questions regarding demography will be included but optional. The impact on

knowledge, values/preferences, involvement, lifestyle, intention to participate, impact on

informed decision making after using the DA will be evaluated quantitively with relevant

questionnaires/items and reported on group level.

***Participants***

Phase 1. The developing process will include lay people aged 23–75, clinical experts, and

other experts (e.g., health care specialists, patient educators, policy makers, psychometry and

IT experts, psychologists) will be approached to be included in the defining scope phase,

steering group, design, alpha- and beta testing. Different individuals will be included in

different phases of the study. Altogether about 20 focus group discussions with 4-6

participants in each group will be performed, and about 100 individual interviews.

Phase 2. The evaluation process will include all individuals invited to breast-, cervix- and

bowel screening programs using the DA and those who use the DA of other interest.

***Analysis***

Qualitative data generated from focus group discussion, individual interviews and

observations will be analyzed using descriptive and manifest content analysis [26].

Quantitative data regarding perceptions of usability, behavior flow, demographics,

knowledge, values, and preferences will be analyzed using relevant descriptive statistical

methods and applicable inferential statistics to assess changes in knowledge, values and

preferences.

***Ethical considerations***

Since seemingly healthy individuals will be approached for the development part of the DA

without any dependent relationship to the researchers, we consider this part of the project

(phase 1) unproblematic from an ethical perspective. During the evaluation process (phase 2)

individuals entering the DA will be approached and asked for participation in the study. Since

participants will respond to questions/statements related to demography, knowledge and

values and preferences, there is a risk for intrusion of integrity. To minimize this risk,

participation will be voluntary, and the DA will be accessible regardless of the individual

choose to participate in the study or not. In addition, personal identification numbers will not

be handled in the current study instead all participants will be provided with a unique code.

Before answering the questions/statements (included in the study), it will be stated that

responding to the questions is equivalent with consenting to the study.

**SIGNIFICANCE**

A DA for cancer screening will gather information to one place on the web and give

individuals the opportunity to elucidate their knowledge, values, and preferences and to

discuss their decision with health professionals. This will lead to more individuals making

autonomous and informed decision in line with their values and preferences including those

known to participate in cancer screening to a lower extent. Altogether leading to more

justified cancer screening programs.

**PRELIMINARY RESULTS**

The DA module for bowel cancer screening, will be based on our previous studies and

relevant scientific literature. The studies include both qualitative and quantitative methods and

are all part of the Screening of Swedish Colons (SCREESCO) study (ID: NCT02078804),

where individuals were randomized to colonoscopy or faecal immunochemical test (FIT). The

aim with SCREESCO was to investigate how CRC screening can impact on incidence and

mortality in CRC. Our studies showed that both participants on non-participants lacked

knowledge about bowel cancer and screening but still had adequate health literacy (HL) and

low levels of anxiety in relation to their decision [19, 27, 28]. However, when studying values

and preferences the groups differed, with non-participants having a more fatalistic approach,

while participants viewed CRC screening as a way of “having control over one’s health” [18,

19]. Regarding different screening methods, we further analysed individuals’ experiences of

the bowel cancer screening procedure (colonoscopy and faecal test). The results describe both

positive and negative emotional reactions, varying burden of the practical part of the

screening procedure, experiences of being inconsistently informed and involved and

expectations not matching reality [29]. To further understand the need of support from health

care professionals we analysed 2100 of 10 000 documented telephone calls (14%) to the

SCREESCO helpline. Unsubscribing or subscribing to screening was the most frequent

reason for calling, followed by organisational issues, counselling, and faecal test problems

(Fritzell et al., 2022 manuscript under review). Counselling regarded mainly abnormal faecal

test results including anxiety related to the result, followed by a need to discuss participation

or not in SCREESCO. The findings will be important to bear in mind when planning and

designing the DA, i.e. what support and information needs individuals invited to CRC

screening in Sweden request.

**REFERENCES**

1. Government Offices of Sweden. *17 sustainable development goals [17 globala mål för*

*hållbar utveckling]*. 210310]; Available from: https://www.regeringen.se/regeringenspolitik/

globala-malen-och-agenda-2030/17-globala-mal-for-hallbar-utveckling/.

2. National Board of Health and Welfare. *Our Work with Agenda 2030 [Vårt arbete med Agenda*

*2030]*. 210310]; Available from:

https://www.socialstyrelsen.se/om-socialstyrelsen/organisation/agenda-2030/.

3. Stacey, D., et al., *Decision aids for people facing health treatment or screening decisions.*

Cochrane Database of Systematic Reviews, 2017(4).

4. Yu, L., et al., *Web-based decision aids to support breast cancer screening decisions:*

*systematic review and meta-analysis.* J Comp Eff Res, 2020. **9**(14): p. 985-1002.

5. Cantor, S.B., et al., *A framework for evaluating the cost-effectiveness of patient decision aids:*

*A case study using colorectal cancer screening.* Prev Med, 2015. **77**: p. 168-73.

6. Coulter, A., et al., *A systematic development process for patient decision aids.* BMC Med

Inform Decis Mak, 2013. **13 Suppl 2**(Suppl 2): p. S2.

7. National Board of Health and Welfare. *Livmoderhalscancer – screening med cytologi och*

*HPV-test*. 2021; Available from:

https://www.socialstyrelsen.se/regler-och-riktlinjer/nationella-screeningprogram/slutligarekommendationer/

livmoderhalscancer/.

8. National Board of Health and Welfare. *Tjock- och ändtarmscancer – screening med test av*

*blod i avföringen*. 2019; Available from: https://www.socialstyrelsen.se/regler-ochriktlinjer/

nationella-screeningprogram/slutliga-rekommendationer/tjock-ochandtarmscancer/.

9. Essink-Bot, M.L. and E. Dekker, *Equal access to colorectal cancer screening.* Lancet, 2016.

**387**(10020): p. 724-6.

10. Zidar, M.N., et al., *Non-attendance of mammographic screening: the roles of age and*

*municipality in a population-based Swedish sample.* Int J Equity Health, 2015. **14**: p. 157.

11. Moustaqim-Barrette, A., et al., *Impact on immigrant screening adherence with introduction*

*of a population-based colon screening program in Ontario, Canada.* Cancer Med, 2019. **8**(4):

p. 1826-1834.

12. Hertzum-Larsen, R., et al., *Participation in cervical cancer screening among immigrants and*

*Danish-born women in Denmark.* Prev Med, 2019. **123**: p. 55-64.

13. Merten, J.W., et al., *Barriers to cancer screening for people with disabilities: a literature*

*review.* Disabil Health J, 2015. **8**(1): p. 9-16.

7

**2022-02-09**

**2022-00786-01-234981**

**Etikprövningsmyndigheten**

Bilaga. Forskningsplan

Kaisa Fritzell, leg. sjuksköterska, Med dr; Anna Jervaeus, leg. sjuksköterska, Docent, Sektionen för

omvårdnad, NVS, Karolinska Institutet

14. Hofmann, B., *Ethical issues with colorectal cancer screening-a systematic review.* J Eval Clin

Pract, 2017. **23**(3): p. 631-641.

15. Honein-Abouhaidar, G.N., et al., *Benefits and barriers to participation in colorectal cancer*

*screening: a protocol for a systematic review and synthesis of qualitative studies.* BMJ Open,

2014. **4**(2): p. e004508.

16. Tatari, C.R., et al., *Perceptions about cancer and barriers towards cancer screening among*

*ethnic minority women in a deprived area in Denmark - a qualitative study.* BMC Public

Health, 2020. **20**(1): p. 921.

17. Azerkan, F., et al., *When Life Got in the Way: How Danish and Norwegian Immigrant Women*

*in Sweden Reason about Cervical Screening and Why They Postpone Attendance.* PLoS One,

2015. **10**(7): p. e0107624.

18. Fritzell, K., et al., *The importance of people's values and preferences for colorectal cancer*

*screening participation.* Eur J Public Health, 2017.

19. Wangmar, J., et al., *Decision-making about participation in colorectal cancer screening in*

*Sweden: Autonomous, value-dependent but uninformed?* Patient Educ Couns, 2020.

20. Nishimura, H., et al., *HPV self-sampling for cervical cancer screening: a systematic review of*

*values and preferences.* BMJ Glob Health, 2021. **6**(5).

21. Yeh, P.T., et al., *Self-sampling for human papillomavirus (HPV) testing: a systematic review*

*and meta-analysis.* BMJ Glob Health, 2019. **4**(3): p. e001351.

22. Moss, S., et al., *Increased uptake and improved outcomes of bowel cancer screening with a*

*faecal immunochemical test: results from a pilot study within the national screening*

*programme in England.* Gut, 2017. **66**(9): p. 1631-1644.

23. Hersch, J., et al., *How information about overdetection changes breast cancer screening*

*decisions: a mediation analysis within a randomised controlled trial.* BMJ Open, 2017. **7**(10):

p. e016246.

24. International Patient Decision Aid Standards IPDAS. Available from: http://ipdas.ohri.ca/.

25. Volk, R.J., et al., *Ten years of the International Patient Decision Aid Standards Collaboration:*

*evolution of the core dimensions for assessing the quality of patient decision aids.* BMC Med

Inform Decis Mak, 2013. **13 Suppl 2**(Suppl 2): p. S1.

26. Elo, S. and H. Kyngäs, *The qualitative content analysis process.* J Adv Nurs, 2008. **62**(1): p.

107-15.

27. Wangmar, J., et al., *Health literacy levels and views about being invited to a colorectal cancer*

*screening program.* Acta Oncol, 2018: p. 1-7.

28. Wangmar, J., et al., *Are anxiety levels associated with the decision to participate in a Swedish*

*colorectal cancer screening programme? A nationwide cross-sectional study.* BMJ Open,

2018. **8**(12): p. e025109.

29. Wangmar, J., et al., *Two sides of every coin: individuals' experiences of undergoing colorectal*

*cancer screening by faecal immunochemical test and colonoscopy.* Eur J Public Health, 2021.
