# Supplementary material for: Making the BEST decision-the BESTa project development, implementation and evaluation of a digital Decision Aid in Swedish cancer screening programmes- a description of a research project
Source: PLoS One. 2023 Dec 12;18(12):e0294332. doi: 10.1371/journal.pone.0294332 (PMC10715660; doi:10.1371/journal.pone.0294332)
Supplement: Attachment — Submitted filename: Responses to reviewer comments_230914.docx [file pone.0294332.s005.docx]

PONE-D-23-16494

Making the BEST decision-the BESTa project

Development and implementation of a digital Decision Aid in Swedish cancer screening programmes- a Study Protocol

PLOS ONE

Dear Dr. Jervaeus,

Thank you for submitting your manuscript to PLOS ONE. After careful consideration, we feel that it has merit but does not fully meet PLOS ONE’s publication criteria as it currently stands. Therefore, we invite you to submit a revised version of the manuscript that addresses the points raised during the review process.
Thank you for the opportunity to review the manuscript. We have done our best to address the points raised during the review process.
==============================
ACADEMIC EDITOR:

Thank you for giving us the opportunity to consider your manuscript. It has now been reviewed and the reviewers' comments are listed below. In particular, one reviewer has expressed considerable criticism of the nature of the article because, even though you declare it to be a study protocol, much basic information is missing and the manuscript as it stands is not sufficient to fully understand what you are doing and how to replicate your intervention elsewhere. Please carefully consider the comments made.
We will consider publishing your manuscript only if you can incorporate the suggestions in a revised version or satisfactorily explain why the comments are not applicable.

==============================

Please submit your revised manuscript by Sep 03 2023 11:59PM. If you will need more time than this to complete your revisions, please reply to this message or contact the journal office at [plosone@plos.org](mailto:plosone@plos.org). When you're ready to submit your revision, log on to <https://www.editorialmanager.com/pone/> and select the 'Submissions Needing Revision' folder to locate your manuscript file.

Please include the following items when submitting your revised manuscript:

- A rebuttal letter that responds to each point raised by the academic editor and reviewer(s). You should upload this letter as a separate file labeled 'Response to Reviewers'.
- A marked-up copy of your manuscript that highlights changes made to the original version. You should upload this as a separate file labeled 'Revised Manuscript with Track Changes'.
- An unmarked version of your revised paper without tracked changes. You should upload this as a separate file labeled 'Manuscript'..

Journal Requirements:

When submitting your revision, we need you to address these additional requirements.

Please ensure that your manuscript meets PLOS ONE's style requirements, including those for file naming. The PLOS ONE style templates can be found at  We have tried our best to adhere to those.
<https://journals.plos.org/plosone/s/file?id=wjVg/PLOSOne_formatting_sample_main_body.pdf> and 
<https://journals.plos.org/plosone/s/file?id=ba62/PLOSOne_formatting_sample_title_authors_affiliations.pdf>.

2. In your Data Availability statement, you have not specified where the minimal data set underlying the results described in your manuscript can be found. PLOS defines a study's minimal data set as the underlying data used to reach the conclusions drawn in the manuscript and any additional data required to replicate the reported study findings in their entirety. All PLOS journals require that the minimal data set be made fully available. For more information about our data policy, please see <http://journals.plos.org/plosone/s/data-availability>. "Upon re-submitting your revised manuscript, please upload your study’s minimal underlying data set as either Supporting Information files or to a stable, public repository and include the relevant URLs, DOIs, or accession numbers within your revised cover letter. For a list of acceptable repositories, please see <http://journals.plos.org/plosone/s/data-availability#loc-recommended-repositories>. Any potentially identifying patient information must be fully anonymized. Important: If there are ethical or legal restrictions to sharing your data publicly, please explain these restrictions in detail. Please see our guidelines for more information on what we consider unacceptable restrictions to publicly sharing data: <http://journals.plos.org/plosone/s/data-availability#loc-unacceptable-data-access-restrictions>. Note that it is not acceptable for the authors to be the sole named individuals responsible for ensuring data access. We will update your Data Availability statement to reflect the information you provide in your cover letter. We have updated this requested information and we hope it is satisfactory.

3. Your ethics statement should only appear in the Methods section of your manuscript. If your ethics statement is written in any section besides the Methods, please move it to the Methods section and delete it from any other section. Please ensure that your ethics statement is included in your manuscript, as the ethics statement entered into the online submission form will not be published alongside your manuscript. This has been corrected.

4. Please include captions for your Supporting Information files at the end of your manuscript, and update any in-text citations to match accordingly. Please see our Supporting Information guidelines for more information: <http://journals.plos.org/plosone/s/supporting-information>. We have updated captions for Supporting information and we hope it has been done as requested.

5. We note that Supporting information Appendix 1 and 2 in your submission contain copyrighted images. All PLOS content is published under the Creative Commons Attribution License (CC BY 4.0), which means that the manuscript, images, and Supporting Information files will be freely available online, and any third party is permitted to access, download, copy, distribute, and use these materials in any way, even commercially, with proper attribution. For more information, see our copyright guidelines: <http://journals.plos.org/plosone/s/licenses-and-copyright>.
We require you to either (1) present written permission from the copyright holder to publish these figures specifically under the CC BY 4.0 license, or (2) remove the figures from your submission:
a. You may seek permission from the original copyright holder of Supporting information Appendix 1 and 2 to publish the content specifically under the CC BY 4.0 license.  
We recommend that you contact the original copyright holder with the Content Permission Form (<http://journals.plos.org/plosone/s/file?id=7c09/content-permission-form.pdf>) and the following text:
“I request permission for the open-access journal PLOS ONE to publish XXX under the Creative Commons Attribution License (CCAL) CC BY 4.0 (<http://creativecommons.org/licenses/by/4.0/>). Please be aware that this license allows unrestricted use and distribution, even commercially, by third parties. Please reply and provide explicit written permission to publish XXX under a CC BY license and complete the attached form.” 
Please upload the completed Content Permission Form or other proof of granted permissions as an "Other" file with your submission.  
In the figure caption of the copyrighted figure, please include the following text: “Reprinted from [ref] under a CC BY license, with permission from [name of publisher], original copyright [original copyright year].”
b. If you are unable to obtain permission from the original copyright holder to publish these figures under the CC BY 4.0 license or if the copyright holder’s requirements are incompatible with the CC BY 4.0 license, please either i) remove the figure or ii) supply a replacement figure that complies with the CC BY 4.0 license. Please check copyright information on all replacement figures and update the figure caption with source information. If applicable, please specify in the figure caption text when a figure is similar but not identical to the original image and is therefore for illustrative purposes only. We have omitted the images.

**Additional Editor Comments:**

Thank you for giving us the opportunity to consider your manuscript. It has now been reviewed and the reviewers' comments are listed below. In particular, one reviewer has expressed considerable criticism of the nature of the article because, even though you declare it to be a study protocol, much basic information is missing and the manuscript as it stands is not sufficient to fully understand what you are doing and how to replicate your intervention elsewhere. Please carefully consider the comments made.
We will consider publishing your manuscript only if you can incorporate the suggestions in a revised version or satisfactorily explain why the comments are not applicable.

**Comments to the Author**

1. Does the manuscript provide a valid rationale for the proposed study, with clearly identified and justified research questions?

The research question outlined is expected to address a valid academic problem or topic and contribute to the base of knowledge in the field.

Reviewer #1: Yes

Reviewer #2: Partly

2. Is the protocol technically sound and planned in a manner that will lead to a meaningful outcome and allow testing the stated hypotheses?

The manuscript should describe the methods in sufficient detail to prevent undisclosed flexibility in the experimental procedure or analysis pipeline, including sufficient outcome-neutral conditions (e.g. necessary controls, absence of floor or ceiling effects) to test the proposed hypotheses and a statistical power analysis where applicable. As there may be aspects of the methodology and analysis which can only be refined once the work is undertaken, authors should outline potential assumptions and explicitly describe what aspects of the proposed analyses, if any, are exploratory.

Reviewer #1: Yes

Reviewer #2: Partly

3. Is the methodology feasible and described in sufficient detail to allow the work to be replicable?

Descriptions of methods and materials in the protocol should be reported in sufficient detail for another researcher to reproduce all experiments and analyses. The protocol should describe the appropriate controls, sample size calculations, and replication needed to ensure that the data are robust and reproducible.

Reviewer #1: Yes

Reviewer #2: No

4. Have the authors described where all data underlying the findings will be made available when the study is complete?

The [PLOS Data policy](https://eur01.safelinks.protection.outlook.com/?url=https%3A%2F%2Fjournals.plos.org%2Fplosone%2Fs%2Fmaterials-and-software-sharing&data=05%7C01%7Canna.jervaeus%40ki.se%7C826747be9a4d4fe8f70108db8f94bd44%7Cbff7eef1cf4b4f32be3da1dda043c05d%7C0%7C0%7C638261641368164617%7CUnknown%7CTWFpbGZsb3d8eyJWIjoiMC4wLjAwMDAiLCJQIjoiV2luMzIiLCJBTiI6Ik1haWwiLCJXVCI6Mn0%3D%7C3000%7C%7C%7C&sdata=e3OV5Q0Vwzndp4xXByPlI3c8plx5rHc80nGOsid6%2FqY%3D&reserved=0) requires authors to make all data underlying the findings described in their manuscript fully available without restriction, with rare exception, at the time of publication. The data should be provided as part of the manuscript or its supporting information, or deposited to a public repository. For example, in addition to summary statistics, the data points behind means, medians and variance measures should be available. If there are restrictions on publicly sharing data—e.g. participant privacy or use of data from a third party—those must be specified.

Reviewer #1: Yes

Reviewer #2: No

5. Is the manuscript presented in an intelligible fashion and written in standard English?

PLOS ONE does not copyedit accepted manuscripts, so the language in submitted articles must be clear, correct, and unambiguous. Any typographical or grammatical errors should be corrected at revision, so please note any specific errors here.

Reviewer #1: Yes

Reviewer #2: Yes

6. Review Comments to the Author

Please use the space provided to explain your answers to the questions above and, if applicable, provide comments about issues authors must address before this protocol can be accepted for publication. You may also include additional comments for the author, including concerns about research or publication ethics.

You may also provide optional suggestions and comments to authors that they might find helpful in planning their study.

**Reviewer #1:** Thank you for the opportunity to review this protocol paper on the development of a digital decision aid for cancer screening in the Swedish context, a timely study given the rising rates of cancer across the globe. There are, however, some gaps that need to be addressed to strengthen the paper:

Background:
• The authors describe the low uptake of cancer screening in general. However, more contextual data is needed about Sweden. What is the current uptake of cancer screening, across cancer programs and demographics? Thank you for this comment. We have expanded with more contextual data as requested (p 3).

• It is unclear how the current screening program works in Sweden. What is meant by ‘region’? Is it a local health department that sends the invitations for cancer screening? This has been clarified in the background section, (pp 3-4).

Methods (pp 7 and onwards)
• The authors focus on the issue of equity and how this digital tool will aim to reduce ongoing inequities in cancer screening. However, more information is needed on how this will be achieved. Thank you, we have tried to address and clarify this relevant issue in different parts of the manuscripts.
o For example, the study will aim to recruit individuals with physical and mental limitations. What are examples of these limitations? Furthermore, how will the study procedures, staff and ultimately, the digital tool accommodate the needs of these participants? Examples of these limitations and possible needs of these participants are now addressed in the manuscript.
o Furthermore, it would be helpful if the authors could expand on the diversity of participants they aim to recruit, whether they will recruit non-English speaking participants and what translation services will be provided if any. Thank you this has been added to the manuscript.
• The authors describe the clinical experts that will be recruited throughout the study. Can they speak on whether any experts in decision-making and digital tools will be included? Yes, we have experts in decision- making and digital tools included in the steering group and possibly also among other experts being approached. It has been added to the manuscript.
• A more in depth description of the qualitative methods that will be used is needed. In particular, it would be helpful if the authors expand on how the data will be analysed and synthesized between the different stages of the study and participants. Additional details on the descriptive and manifest content analysis that is mentioned is also needed. This has been clarified in the manuscript.
• 33 participants will be included for each screening program interview study. How was this number selected? Thank you for this comment. We realize that this has been confusing since it is difficult to provide an exact number of individuals in a qualitative research context. Therefore, this section has been re-written to clarify how number of participants are decided upon in qualitative research and in usability testing. Please also see answer to Reviewer #2 comment in the end of this document.
• The flow diagram in the appendix would benefit from additional details such as the number of participants included at each stage. This is difficult to provide since data saturation is what direct number of participants needed at each stage.

Discussion (pp 15 and onwards)
• The authors mention that patients and the lay public are involved in the study. However it is a little unclear about whether this is related to the direct study participation or actual design of the protocol. We have lay people involved in both the design of the protocol and in the study itself (the three phases). This has been clarified.
• Some details around how long will the decision aid take users to complete would be very helpful. This is difficult to answer since the decision aid is not considered as something to complete, rather as an information and interactive tool. A sentence regarding this has been added to the manuscript.

Other
• There is a small typo in Appendix 1- should be ‘alpha’ not ‘alfa’. Thank you, we have corrected this.

**Reviewer #2:** Although I have concluded that a major revision is required, I am still rather mystified by the tittle including the word ‘Protocol’. Perhaps what is needed is a rethink as to the purpose of this paper?

Simon Day defined a Protocol as “written document describing all the important details of how as study will be conducted. It will generally include details of the product’s being used, rationale for the study, what procedures will be carried out on subjects in the study, how many subjects will be studied, the design of the study and how the data will be analysed”

Clearly although this paper is not describing a clinical trial, it is allegedly describing a study which will require substantial and detailed blocks of activities requiring how and when the various components will be implemented. So, a protocol is more like an instruction manual which the current paper clearly is not. Thank you for this comment. This paper is a *description* on the design, implementation and evaluation of a digital decision aid and may not be considered a protocol as defined in the comment or elsewhere. Our described procedures are based on the IPDAS framework [1, 2] and the model development process for DAs presented by Coulter et al., [3] i.e. the process is well-documented and researched. The outline of the manuscript has been inspired by work on development of digital decision aids for similar purposes in both Sweden and the Netherlands [4-6]. We understand that this has been confusing and therefore we have changed the title and made an effort to adapt and clarify in the manuscript accordingly.

From a statistical perspective there is, for example, no indication of how many subjects will be interviewed at the various stages, nor of how the overall assessment of the value of the programme will be made. Thank you for this comment. We have clarified on how the recruitment procedure will take place, and since data collection in Phase 1 (Description of the process and implementation evaluation) will be based on interviews and focus group discussions it is less applicable to describe number of subjects to include. In the previous version we gave an estimate on number of participants, based on our previous qualitative research, but have now instead added a paragraph on data saturation, suitable when handling qualitative data and a paragraph on number of users to identify problems in usability testing (pp 10-11). For phase 2 (Description of the outcome evaluation) number of subjects is dependent on number of individuals entering the iDA and who further consent to participate in the evaluation. Regarding the overall assessment of the value of the programme this will be done through the self-reported questionnaires where individuals can indicate the value of the iDA and if they have any suggestions for improvements etc. We have clarified this in the manuscript.

References:

1.	International Patient Decision Aid Standards IPDAS. Available from: <http://ipdas.ohri.ca/>.

2.	Elwyn, G., et al., *Developing a quality criteria framework for patient decision aids: online international Delphi consensus process.* Bmj, 2006. **333**(7565): p. 417.

3.	Coulter, A., et al., *A systematic development process for patient decision aids.* BMC Med Inform Decis Mak, 2013. **13 Suppl 2**(Suppl 2): p. S2.

4.	Woudstra, A.J., et al., *Development and pilot-testing of a colorectal cancer screening decision aid for individuals with varying health literacy levels.* Patient Educ Couns, 2019. **102**(10): p. 1847-1858.

5.	Grim, K., et al., *Development and usability testing of a web-based decision support for users and health professionals in psychiatric services.* Psychiatr Rehabil J, 2017. **40**(3): p. 293-302.

6.	Kjörk, E.K., et al., *Development of a Digital Tool for People With a Long-Term Condition Using Stroke as a Case Example: Participatory Design Approach.* JMIR Hum Factors, 2022. **9**(2): p. e35478.
